# Inferring Protein Modulation from Gene Expression Data Using Conditional Mutual Information

**DOI:** 10.1371/journal.pone.0109569

**Published:** 2014-10-14

**Authors:** Federico M. Giorgi, Gonzalo Lopez, Jung H. Woo, Brygida Bisikirska, Andrea Califano, Mukesh Bansal

**Affiliations:** 1 Department of Systems Biology, Columbia University, New York, New York, United States of America; 2 Center for Computational Biology and Bioinformatics, Columbia University, New York, New York, United States of America; 3 Columbia Genome Center, High Throughput Screening facility, Columbia University, New York, New York, United States of America; 4 Department of Biomedical Informatics, Columbia University, New York, New York, United States of America; 5 Department of Biochemistry and Molecular Biophysics, Columbia University, New York, New York, United States of America; 6 Institute for Cancer Genetics, Columbia University, New York, New York, United States of America; 7 Herbert Irving Comprehensive Cancer Center, Columbia University, New York, New York, United States of America; University of Manchester, United Kingdom

## Abstract

Systematic, high-throughput dissection of causal post-translational regulatory dependencies, on a genome wide basis, is still one of the great challenges of biology. Due to its complexity, however, only a handful of computational algorithms have been developed for this task. Here we present CINDy (Conditional Inference of Network Dynamics), a novel algorithm for the genome-wide, context specific inference of regulatory dependencies between signaling protein and transcription factor activity, from gene expression data. The algorithm uses a novel adaptive partitioning methodology to accurately estimate the full Condition Mutual Information (CMI) between a transcription factor and its targets, given the expression of a signaling protein. We show that CMI analysis is optimally suited to dissecting post-translational dependencies. Indeed, when tested against a gold standard dataset of experimentally validated protein-protein interactions in signal transduction networks, CINDy significantly outperforms previous methods, both in terms of sensitivity and precision.

## Introduction

Reverse engineering of gene regulatory networks using gene expression profiles has proven valuable in dissecting the logic of cellular regulation in multiple species [1]–[4] and in elucidating mechanisms governing pathophysiological processes [5]–[7]. However the vast majority of these methods has been developed for the dissection of pairwise relationships between gene-products, for instance by using co-expression [8], information theoretic [3], and Bayesian Network [9] methods. These are well-suited to identify relatively static interactions between transcription factors (TFs) and targets or protein-protein interactions (PPIs) in complexes [10] but fail to capture the more complex dynamic rewiring of regulatory interactions implemented by signal transduction, post-transcriptional regulation, and multi-TF combinatorial regulation. However, most regulatory dependencies, such as regulation of target expression by a TF, are not static but rather depend on additional events, such as the availability of co-factors and microRNAs or on protein modification events such as acetylation, phosphorylation and ubiquitylation, which dynamically rewire the logic of the cell in response to specific exogenous and endogenous signals [11].

**Figure 1:**
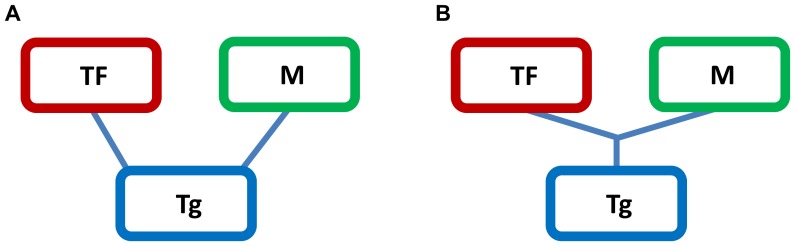
Alternative three-way network topologies including a Transcription Factor (TF), a Target gene (Tg) and a Modulator gene (M). (A) depicts the independent regulation of the target gene by a modulator and a TF; (B) describes a three-way interaction between the TF, the target gene and the modulator.



**Figure 2:**
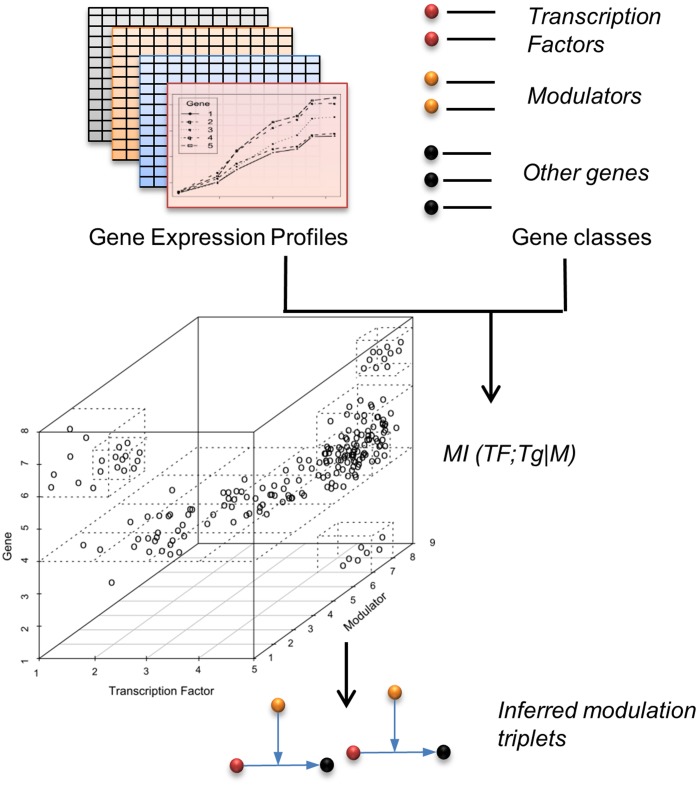
Schematic representations of the CINDy algorithm. A collection of gene expression profiles is required to calculate Conditional Mutual Information between lists of modulators, transcription factors and putative target genes, with the final output of inferred modulation events.

These observations provided the original rationale for the development of the Modulator Inference by Network Dynamics (MINDy) algorithm [12]. MINDy was instrumental in the elucidation of novel modulators of oncogene TF activity, such as the STK38 kinase and the HUWE1 ubiquitin ligase as regulators of MYC and MYCN ubiquitin dependent proteasomal degradation, respectively, which were experimentally validated [6], [13]. MINDy relied on information theoretic principles to identify candidate modulators of TF activity, specifically by assessing the difference in mutual information, 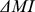, between a TF and its target genes, when conditioning on the highest and lowest expression of any candidate modulator gene [14]. The algorithm was very effective in predicting novel candidate modulators that could be experimentally validated and associated with regulation of specific post-translational modifications [6], [12], [13], [15]. However, it was never systematically tested across a comprehensive set of established post-translational dependencies and suffers from a relatively high false negative rate. Indeed, use of the 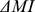 was originally chosen as a heuristic approximation of the theoretically correct analytical formulation. This analytical formulation analyzes the differences in multi mutual information of two different distributions describing two different topologies, one depicting the independent regulation of a target gene (Tg) by a modulator (M) and a TF (**Figure 1A**) and the second one depicting a three-way interaction between the TF, the target gene and the modulator (**Figure 1B**). As proposed in [12], this difference requires estimation of:
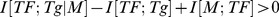
(Eq.1)for the inference of a three-way interaction, where M is any modulator protein affecting the ability of a transcription factor (TF) to regulate its targets (Tg). Indeed, at the time the algorithm was developed, using the theoretically derived formulation would have been a prohibitive undertaking, both computationally and in terms of data requirements. One of the critical limitations of the 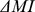 heuristic was that we had to assume 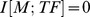, thus limiting the analysis strictly to modulators whose expression was statistically independent of the TF's, a condition that precluded the analysis of many relevant modulator proteins. This constraint limits the inference of three-way interactions to the conditional interactions, *i.e.* those between TF and Tg that are conditionally dependent on the expression of M. Inference of true conditional transcriptional interactions requires.

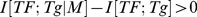
(Eq.2)Moreover, the explicit test of independence (i.e. 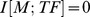) increases the false negative rate by not considering the possibility where despite the existence of dependency between M and TF's expression, Eq. 2 is satisfied. To address these problems we now introduce a computationally efficient solution to estimate the full conditional mutual information (CMI), based on adaptive partitioning [16], thus avoiding any heuristics, removing the limitations of the previous formulation, and embracing the correct theoretical model for the dissection of conditional interactions. Adaptive partitioning is a very efficient method for calculating the Shannon entropy of joint gene distributions [16], using a histogram based approach (**Figure 2**). The new approach has been implemented in a novel algorithm for the Conditional Inference of Network Dynamics (CINDy). Elucidating candidate modulators of TF activity is an extremely important problem in biology, as it helps dissect the logic by which signal transduction pathways regulate transcriptional programs. We applied CINDy to two independent datasets and evaluated its precision and sensitivity in predicting experimentally validated post-translational modulators of TF activity. We also compared the performance of CINDy with the original MINDy algorithm. There are virtually no other available algorithms to dissect post-translational dependencies from gene expression profile data. As a result, comparison to MINDy is the most appropriate for the new algorithm.

## Results

First, we tested the performance of the two algorithms (using default parameters) in inferring established modulatory interactions, using two distinct gene expression profile datasets: (a) a B-cell lymphoma dataset containing 226 samples [17] and (b) a lung adenocarcinoma TCGA dataset containing 412 samples [18]. These datasets were specifically selected to evaluate the algorithms' performance and applicability within different contexts and using gene expression profiles from different platforms (Affymetrix U133P2 microarrays and RNASeq, respectively). The results of these analyses are summarized in **Table 1**.

**Table 1:** Default parameters used for running MINDy.

	Parameters				
	Percentage of samples in each tail	MI p-value for independence between modulator and transcription factor	Corrected pvalue threshold for each modulator, transcription factor and target interaction	MI p-value for independence between transcription factor and target	FDR p-value for TF/modulator pair
MINDy	35%	10^−5^	0.05	10^−6^	0.05
CINDy	NA	NA	0.05	NA	0.05

NA: Not applicable.

Briefly, for the MINDy algorithm, for each candidate modulator gene, M, we tested only TFs with expression statistically independent of M, as assessed by the statistical significance of the Mutual Information of their gene expression profiles. We also discarded candidate target genes whose gene expression was highly correlated with that of the associated TF, thus restricting the number of candidate target genes in the analysis. Both of these are a requirement for using the 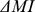 heuristics in place of the full CMI formulation. MINDy proceeds by selecting two non-overlapping sample subsets (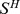 and 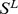) representing 35% highest and 35% lowest expression of M (a heuristically selected threshold). Then, for each TF considered in the analysis, the mutual information 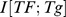 between the TF and each candidate target gene is computed independently from the 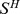 and from the 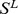 samples and the statistical significance of their difference (i.e., 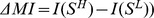 is evaluated using a null model based on sample permutations. For each candidate M 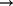 TF interaction, the number of target genes, 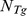, producing a statistically significant 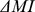 is computed. For CINDy, instead, the full conditional mutual information analysis is performed (see Eq. 2 and **Materials and Methods**).


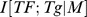
 is calculated using an estimation of 3-dimensional probability distribution, whereas 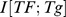 is calculated using an estimation of 2-dimansional probability distribution, therefore numerically 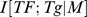 cannot be compared to 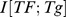, thus making the calculation of Eq.2 a non-trivial problem. To solve this, we used a null model that is centered around 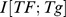 (see **Materials and Methods**). This null model not only eliminates the need to compare 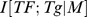 and 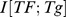 but also assesses the statistical significance of Eq. 2. This eliminates both the 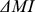 heuristics, as well as the arbitrary parameter controlling the tail sizes used in the MINDy implementation. Again, the number of candidate targets 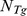 needed to produce a statistically significant CMI for a candidate M 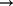 TF interaction is computed. Finally, for both algorithms, significant M 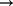 TF interactions are inferred based on the number of statistically significant conditional target interactions, using a statistical model. In brief, for a particular FDR threshold (default FDR = 0.05), the number of affected targets in the null hypothesis is assessed by running both algorithms repeatedly over the same dataset, following random modulator expression assignment. The final result is a list of M 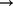 TF pairs and associated p-values.

To objectively assess the performance of the two algorithms, we compared the modulatory interactions they inferred to a set of validated Protein-Protein Interactions (PPIs) between TFs and candidate modulator proteins (“gold standard dataset,” PPI_Gold_). The latter was generated by taking the union of interactions obtained from four independent databases: for generic PPIs, we combined the interactions in HPRD [19], Y2H db [20] and STRING [21], while for candidate kinase/target pairs, we used the PhosphoSite database [22] (see **Materials and Methods** and **Figure S1**). Algorithm performance was evaluated by computing recall rate, defined as the fraction of inferred interactions in the PPI_Gold_, and precision rate, defined as 1 minus the fraction of inferred interactions not present in the PPI_Gold_. An important point to note is that the PPI_Gold_ dataset contains only a very small fraction of all true biological PPIs. Therefore, any precision estimates represent highly underestimated values. Indeed, precision should be used only as a comparative metric here such that recall may be computed either at roughly the same or better precision and is not representative of true precision, which can only be assessed from experimental validation.

**Figure 3:**
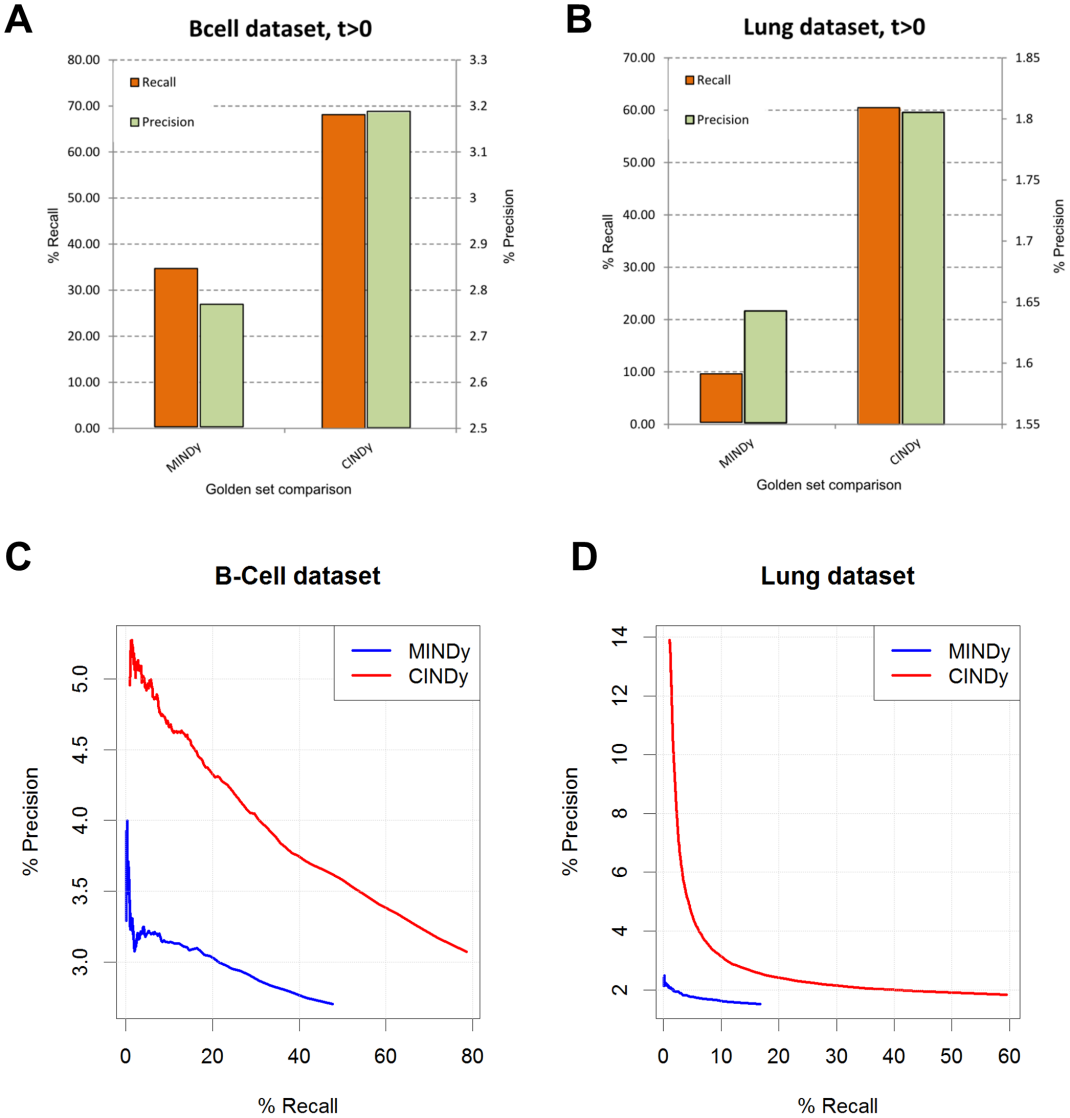
Comparative performance of MINDy and CINDy. Precision and recall values are compared in the B-cell lymphoma dataset (panel A) and Lung Adenocarcinoma dataset (panel B), calculated by matching the predictions with a gold standard dataset set obtained from four different databases of experimentally validated PPIs between modulators and transcription factors. Precision and recall are further compared at different robustness threshold for MINDy (blue line) and CINDy (red line) in the B-cell dataset (panel C) and in the Lung dataset (panel D, see **Materials and Methods**).

In B-Cell lymphoma (**Figure 3A**), CINDy outperformed MINDy by achieving significantly higher recall and precision. In fact, CINDy achieves roughly twice the recall of MINDY (68.13% vs. 34.37%) while also increasing precision (3.19% vs. 2.76%). Similarly, in lung adenocarcinoma (**Figure 3B**) CINDy achieves a 60.50% recall rate with 1.81% precision, whereas MINDy achieves a dramatically smaller recall of 9.26% at an even lower precision of 1.64%.

We also evaluated the performance of both algorithms by changing the stringency of the analysis, i.e., the minimum number of statistically significant (TF, Tg) interactions (i.e., 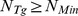) required to call a M 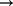 TF modulatory interaction. To simplify the analysis, since the number of significant target interactions is discrete and hence a precise relationship with a meaningful FDR rate is not always possible, we considered 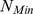 values between 1 and 300, a significant FDR range between 1 and 10^−16^. As expected, with the increase of the stringency threshold we observed a decrease in recall rate by both methods in both datasets (**Figure 3C–D**). However, at any equivalent recall rate, CINDy significantly outperformed MINDy. As expected, precision was positively correlated with the stringency threshold. Taken together, these findings show that use of the correct conditional mutual information model significantly outperforms the 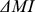 heuristics proposed in [2].

Due to the resulting differences in the analysis, the computational requirements of the algorithms are different (**Figure S2**). When using identical computational environments, CINDy requires almost double the time of MINDy, mostly due to using the entire dataset rather than just the top and bottom 25%. However, its memory requirements are half of those of MINDy (**Figure S2**).

Much of the computational requirements for the algorithms are due to the fact that every gene is considered as a candidate TF target by the analysis. To reduce both computation time and memory requirements (**Figure S3**), one can consider TF targets that are either experimentally assessed from CHIP-Seq and or TF silencing assays [23], [24], inferred by reverse engineering algorithms, such as ARACNe [3], CLR [25], Mider [26], and others [27]–[29], or from sequence specific TF binding sites [30]. Although this provides a significant computational advantage and without decreasing precision, use of pre-determined target genes significantly decreases the number of correctly predicted TF modulators in the gold set, hence increasing the false negative rate (**Figure S4** and **Table S1**).

Finally, we assessed the performance of CINDy by varying the number of gene expression profiles, *n*. We varied *n* from 50 to 200 with an interval of 25 and assessed the performance by inferring modulatory interactions for 100 transcription factors and modulators with maximum connection in the gold standard dataset. For a given *n* we repeated the assessment 100 times by resampling the gene expression profiles. This analysis showed that whereas there is no change in the precision with varying *n* there is a constant increase in recall rate with increasing *n* (**Figure S5**).

### CINDy identifies novel modulatory interactions

**Figure 4:**
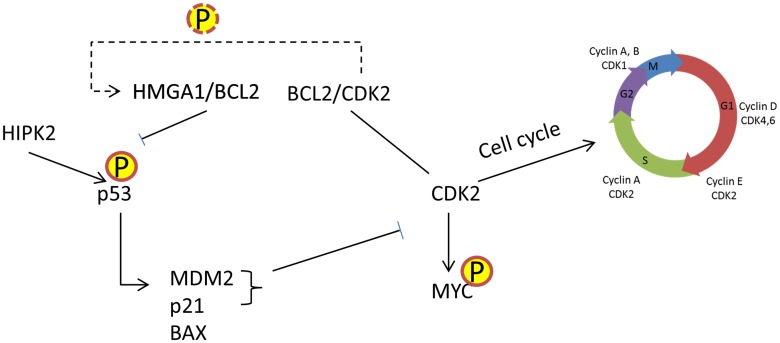
Example of novel prediction by CINDy. Proposed mechanism for modulation of HMGA1 by CDK2.

CINDy confirmed previous predictions of modulatory interactions, such as MYC activity modulation by the STK38, MAPK1 and CSNK2A1 proteins in B-cell lymphoma [12], but it also inferred a large number of established post-translational regulatory interactions that could not be detected by MINDy (**Table S2**), as well as several novel predictions. Among the newly inferred MYC activity modulators, we find many signaling proteins and TFs that are associated with B-lymphoma malignancies, including ATM [31], CDK2 [32], MYC [33], [34], HIF1A [35] [36] and NFKB [37]. In addition, many of the protein pairs inferred only by CINDy are well-known and have been experimentally validated, e.g. GSK3B/MYC [38], IKBKB/NFKB1 [39], MAPK1/MYC [40]. However, when considering post-translational modulators of proteins known to play a causal role in B-lymphoma, such as MYC and BCL6, their CINDy inferred modulators are generally unknown and likely to be experimentally validated, since experimental validation of MINDy prediction has been consistently in the 70% - 80% range. These predictions identify several interesting and potentially biologically relevant links. For instance, the interaction between CDK2 and HMGA1, predicted only by CINDy, may constitute a previously uncharacterized signaling bridge during cell cycle progression. The CDK2 kinase belongs to the family of cyclin-dependent kinases (CDKs) regulating cell cycle [41] and its activity depends on the interactions with other regulatory proteins, A or E-type cyclins, complexes of which are involved in the regulation of G1 and S phase transitions [42], [43]. The functional role of CDK2 in maintaining neoplastic growth was previously reported [44]–[46]. HMGA1 belongs to the family of non-histone chromatin-associated high-mobility group proteins involved in various cellular processes including heterochromatin organization, regulation of gene transcription, DNA replication and it is overexpressed in malignant neoplasms but not in normal adult cells [47]. Causal regulation of HMGA1 activity by CDK2 was never previously reported. However, there are many clues suggesting that such an interaction may be realistic (**Figure 4**). HMGA1 was shown to contribute to neoplastic transformation by modulating transcriptional activity of p53 leading to inhibition of apoptosis [48], [49]. Transcriptional targets of p53, MDM2 and p21, have been shown to inhibit CDK2 activity and contribute to p53-dependent cell cycle arrest [50]. Both, HMGA1 and CDK2 were shown to interact with BCL2 [51], [52]. Hence it is not unlikely that they may form a functional complex. Thus MINDy provides direct clues leading to experimentally testable hypotheses that may elucidate novel functional interactions in tumorigenesis as previously reported [2], [6], [13].

In lung adenocarcinoma, CINDy specifically highlights modulatory interactions that affect epithelial proliferation, such as the direct phosphorylation by the Epidermal Growth Factor Receptor (EGFR) of the STAT1 [53] and STAT3 [54] TFs, a fundamental and well established step in the proliferative signal transduction cascade that was not detected by MINDy. Another modulatory interactions found exclusively by CINDy is the phosphorylation of GATA binding protein 1 (GATA1) by the kinase ERK2/MAPK1 [55]. Other non-phosphorylation dependent modulations, like the transcriptional co-activation of the proliferative transcription factor Forkhead box protein M1 (FOXM1) by the histone acetyltransferase CREB binding protein (CREBBP), show how the algorithm can dissect a variety of regulatory interactions, mediated by diverse post-translational mechanisms and simply undetectable by conventional gene expression analysis [56].

## Discussion

The most pressing challenge for Systems Biology is the development of model-based approaches for the veritable interpretation of an avalanche of new biological data. Reverse engineering algorithms provide a key approach to build regulatory models representing the molecular mechanisms that control cell behavior. These models in turn can provide critical novel knowledge about mechanistic control of physiologic processes [57] and their dysregulation in disease [5]–[7], [58]–[62], thus allowing the rapid, genome-wide generation of new testable biological hypotheses. By leveraging broadly available gene expression data, the MINDy algorithm allowed high-fidelity reconstruction of complex post-translational causal dependencies, where a modulator protein can affect the transcriptional activity of a TF on its targets. Replacing the original empirical formulation of the MINDy algorithm with the theoretically correct one, based on the conditional mutual information, the CINDy algorithm dramatically improves both recall and precision, thus virtually doubling the number of candidate modulatory interactions while also decreasing false positives. This allows inference of many interactions that were experimentally established such as the activation of the STAT TFs by EGFR aberrant signals or the activation of MYC by GSK3B, which could not be previously detected. The inclusion of prior knowledge to reduce the search space of CINDy to a subset of the potential TF target genes shows benefit both in terms of increased precision and substantial decrease in computational time, albeit at the price of decreased sensitivity. The dataset size also seems to be affecting the performance of CINDy, since intuitively, more samples drive higher recall rates at comparable precision. Fewer than 100 samples results in very small recall rate and only by using>150 samples does CINDy produce a reasonable recall rate (>20%). Therefore, it is recommended to use CINDy with a minimum of 150 samples. It is foreseeable that in the future with the concurrent increase of broader and more accurate databases for context-specific experimentally validated regulatory networks, more sophisticated CMI-based tools will be developed to integrate weighted evidences coming from different sources, such as novel MI-based reverse engineering methods [63], sequence motif analysis [64], or ChIP-seq data [23]).

Due to its general formulation, CINDy can identify a variety of post-translational interaction mechanisms that go beyond standard post-translational modification (e.g., phosphorylation, or ubiquitylation events), such as recruitment of CREBBP to FOXM1 and consequent transcriptional activation. It is also able to generate novel testable hypotheses for intriguing dependencies, such as regulation of HMGA1 activity by CDK2 (**Figure 4**).

Importantly, by adopting a theoretically rigorous formulation, CINDy does away with many of the heuristics and parameter choices of the MINDy implementation. For instance, the need to select arbitrary tails of the modulator expression, the somewhat arbitrary thresholds used to evaluate a modulator TF interaction or the statistical dependency between a TF and a candidate target gene, as well as the statistical significance of 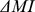 (**Table 1**). CINDy effectively eliminates the requirement to choose nonstandard values for these parameters or eliminates them altogether. Indeed, CINDy requires only the selection of a statistical threshold to evaluate the statistical significance of the CMI, thus making the algorithm extremely robust. Altogether, our finding shows that CINDy is a novel standard tool for inferring genome-wide modulation events affecting transcription factor activity.

## Materials and Methods

### Expression datasets

We ran the CINDy and MINDy algorithms on two independent datasets, called “Lung dataset” and “B-Cell dataset”. The Lung dataset originates from the TCGA gene expression study [18], and it contains genome wide gene expression profiles of 412 RNASeq samples (Synapse v6 release: https://www.synapse.org/#!Synapse:syn395683), RPKM-normalized. The B-Cell dataset derives from human B-cell microarray gene expression experiments [17], and it's constituted by 226 samples profiled on the Affymetrix U133P2 platform.

### Transcription factors and modulator genes

Transcription factors and modulator gene lists used to run MINDy in this study were defined as in [12] and then further extended with the current Gene Ontology (GO) annotations [65]. In brief, a “transcription factor” gene was defined as such if annotated in the GO molecular category “transcription factor activity”, while a “modulator” is defined as a gene belonging to any of the following molecular functions: protein kinase activity, phosphoprotein, phosphatase activity, acetyltransferase activity, deacetylase activity or signal transduction. The lists were further manually curated and are available in the **Table S3**, containing 3,203 candidate modulators and 1,673 transcription factors. 210 of these genes fall in both categories (e.g. CREB1, NFKB1 and TP53), i.e. they have both the transcriptional as well as modulatory function, and were therefore processed in our analyses both as candidate modulators and transcription factors.

### Gold standard sets

We collected human PPI interactions from HPRD release 9 (3,637 unique modulator/TF interactions), Y2H (170), Strings v9.0.5 (81,504) and human phosphorylation kinase/target pairs from PhosphoSite (541), totaling 82,160 distinct modulator/TF interactions (**Figure S1**). We excluded homodimerization interactions and peptides that could not be unambiguously mapped to any Entrez gene id.

### Adaptive Partitioning (AP)

AP is an algorithm for dynamic binning of the expression distribution, which can be applied for calculation of mutual information between two or more variables [16], [26]. An initial partitioning is applied, centered on the median of the distributions, and then partitioning proceeds in the quadrants where the sample distribution is significantly non-uniform (assessed by 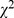 test)

### Conditional Mutual Information (CMI)

The CMI between a Transcription Factor (TF) and a Target Gene (Tg), given a putative Modulator (M) is inferred by estimating the conditional probability distribution using an adaptive partitioning approach:
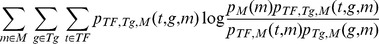
(Eq.3)where p indicates the outcome probability for a given gene expression range.

CMI is therefore analogous to conditional partial correlation for mutual information calculation [66]: the relationship between TF and Tg is assessed while keeping M constant. If this relationship changes significantly depending on the M distribution, MINDy will report M as a putative modulator of the interaction between TF and Tg (**Figure 2**).

### Null Model to estimate significance of CMI

To assess the statistical significance of a particular CMI, we generate a series for null models, each for different ranges of mutual information between TF and Tg. To build this null model, first we randomly select 10^4^ distinct (TF,Tg) pairs and estimate 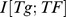 between them using the adaptive partitioning method. Next for each of these pairs we calculate 1000 CMI scores using the randomized expression of modulators. We bin the entire range of 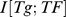 into 100 equi-probable bins, resulting in 100 TF-Tg pairs and 10^5^ CMI values in each bin. Within each bin, we model the distribution of CMI as an extended exponential, 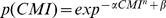 (as described in [67]). To estimate the pvalue of given CMI, we estimate the mutual information between TF and Tg from this CMI to identify the bin and use the extended exponential model from that bin to extrapolate the probability of that CMI.

## Supporting Information

Figure S1 **Number of modulator/transcription factor associations in four independent databases, and relative intersections.**
(TIF)

Figure S2 **Comparative computational performance of MINDy and CINDy.** The test was performed on the human B-cell dataset [17] with 100TFs 100 Modulators and 250 samples. Reported are the mean and standard deviations of all the 100 MINDy runs. The performance was assessed on a 16 x Intel Xeon CPU E5-2630 0 @ 2.3 GHz machine with 30,098,316K total RAM.
(TIF)

Figure S3 **CINDy performance on a single TF-Modulator pair using increasing number of target genes.** The vertical black line to the left indicates the average number of targets in the dataset (97.2). For this particular dataset, on average, MINDY using all genes is almost 130 times slower than using target genes, and requires almost 28 times more RAM.
(TIF)

Figure S4 **Benchmark of MINDy runs using a subset of target genes defined by ARACNe** [27] **(p-value 10e-8).** A-B Precision and recall of MINDy, CINDy, intersection and union sets in the B-cell and Lung datasets, calculated over a golden set of four databases of experimentally validated PPIs between modulators and transcription factors. C-D Precision/Recall plots for MINDy (blue points) and CINDy (red points) at different robustness thresholds (see **Materials and Methods**).
(TIF)

Figure S5 **Effects of sample size on precision and recall in the B-cell dataset (226 samples).** The precision/recall curves were calculated using the 100 TFs and modulators with most connections in the gold standard set (**Figure S1**). The error bars indicate the standard deviation in the estimation of precision and recall obtained by running CINDy over 100 datasets generated by subsampling.
(TIF)

Table S1 **Raw performance information for CINDy and MINDy in the lung adenocarcinoma and B-cell lymphoma datasets, at different thresholds defined by the number of target genes affected by the modulation events.**
(XLSX)

Table S2
**Significant modulation events predicted by CINDy and MINDy at standard parameters in the B-Cell and Lung datasets.** The number of significant conditional target interactions for each M 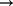 TF is also reported.(XLSX)

Table S3 **Gene symbols used in the current manuscript as modulator genes or transcription factors.**
(XLSX)
