# Correction: Inferring Protein Modulation from Gene Expression Data Using Conditional Mutual Information

**DOI:** 10.1371/journal.pone.0163402

**Published:** 2016-09-15

**Authors:** Federico M. Giorgi, Gonzalo Lopez, Jung H. Woo, Brygida Bisikirska, Andrea Califano, Mukesh Bansal

The following information is missing from the Acknowledgments section: The results published here are in part based upon data generated by the Therapeutically Applicable Research to Generate Effective Treatments (TARGET) initiative managed by the NCI. The data used for this analysis (phs000218.v15.p5.c1) are available at http://www.ncbi.nlm.nih.gov/projects/gap/cgi-bin/study.cgi?study_id=phs000218.v15.p5. Information about TARGET can be found at http://ocg.cancer.gov/programs/target.
