# Supplementary figures and images for: Inferring Protein Modulation from Gene Expression Data Using Conditional Mutual Information
Source: PLoS One. 2014 Oct 14;9(10):e109569. doi: 10.1371/journal.pone.0109569 (PMC4196905; doi:10.1371/journal.pone.0109569)

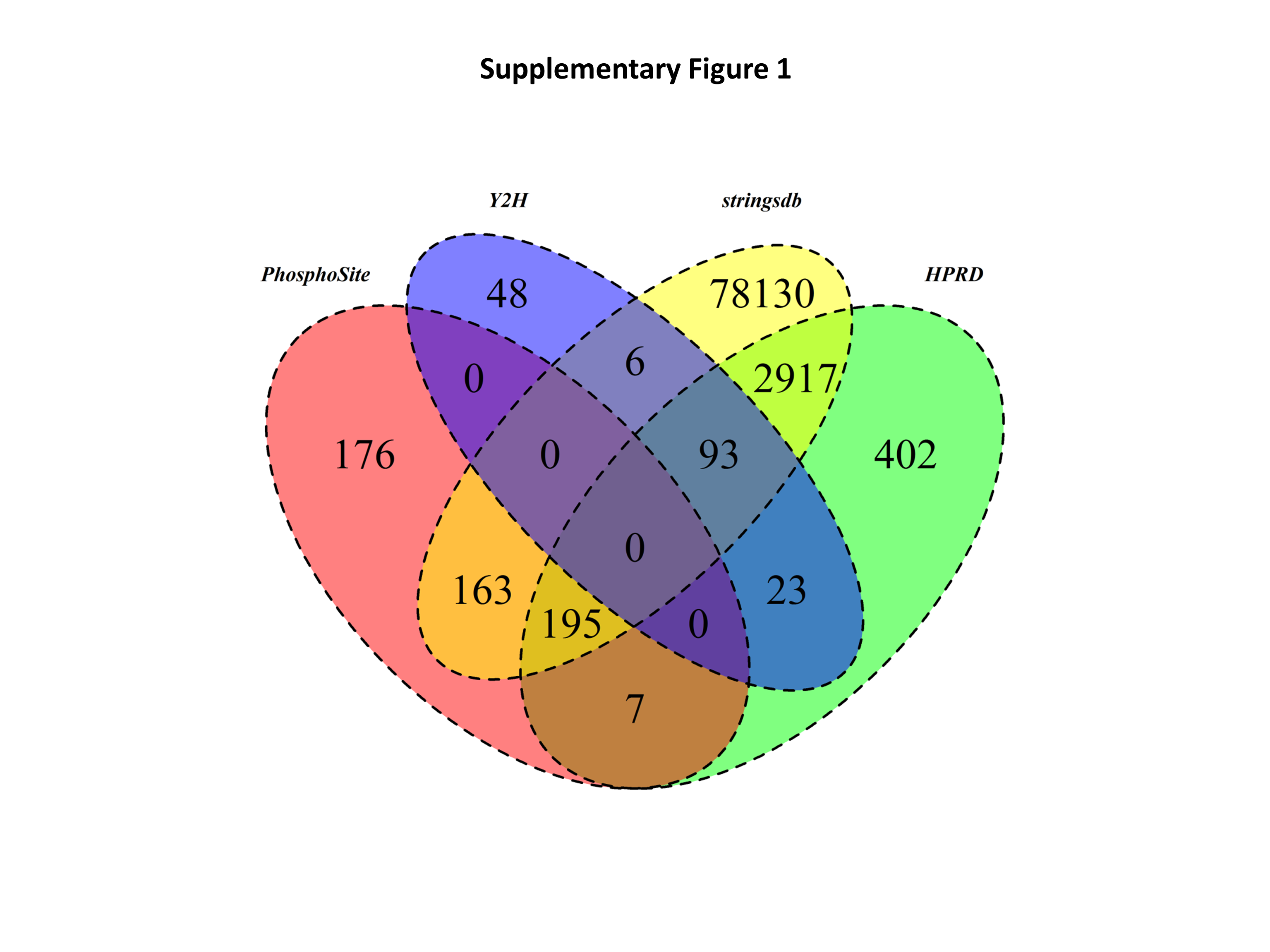

Supplement: Figure S1 — Number of modulator/transcription factor associations in four independent databases, and relative intersections. (TIF) [file pone.0109569.s001.tif]

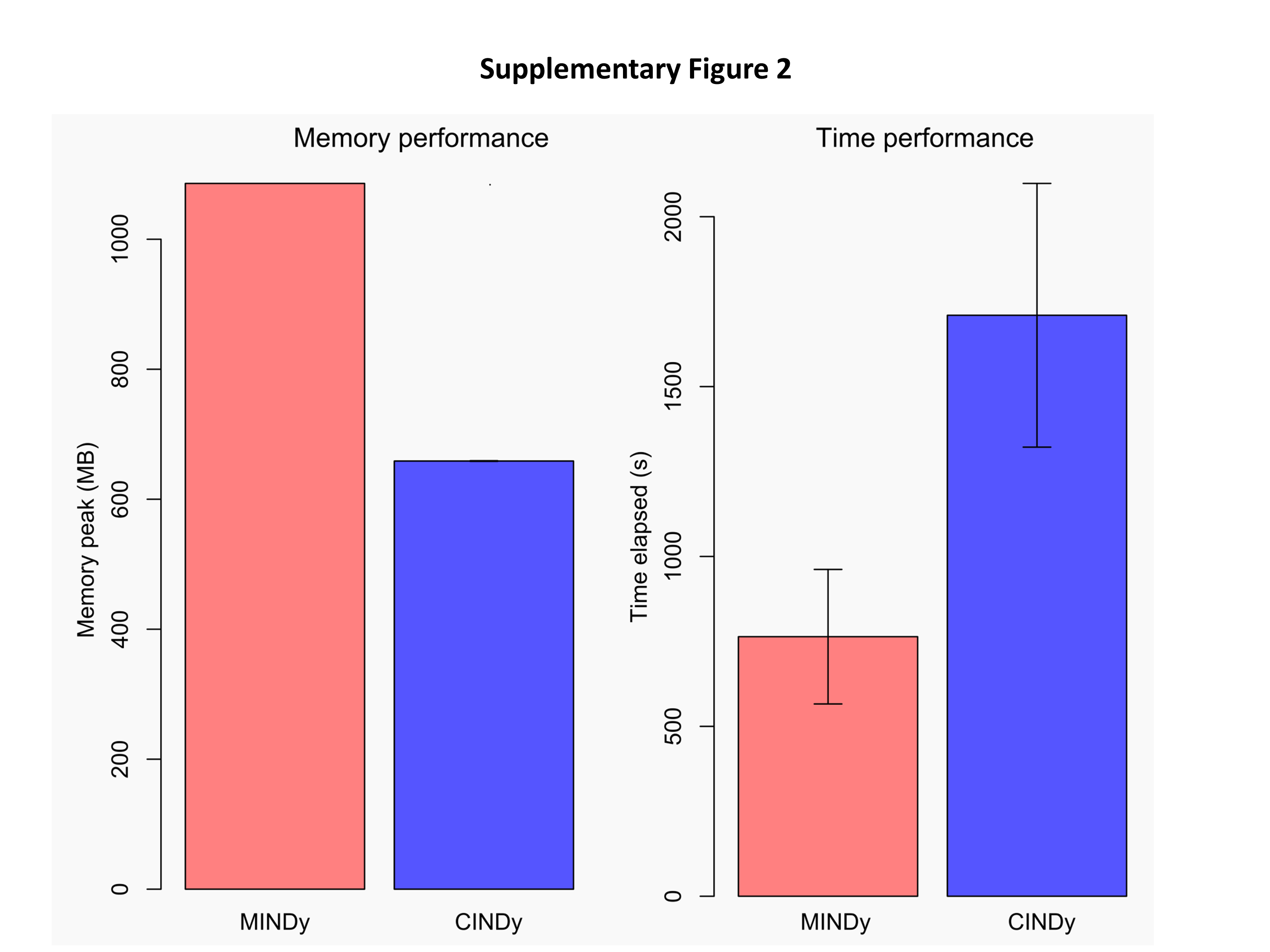

Supplement: Figure S2 — Comparative computational performance of MINDy and CINDy. The test was performed on the human B-cell dataset [17] with 100TFs 100 Modulators and 250 samples. Reported are the mean and standard deviations of all the 100 MINDy runs. The performance was assessed on a 16 x Intel Xeon CPU E5-2630 0 @ 2.3 GHz machine with 30,098,316K total RAM. (TIF) [file pone.0109569.s002.tif]

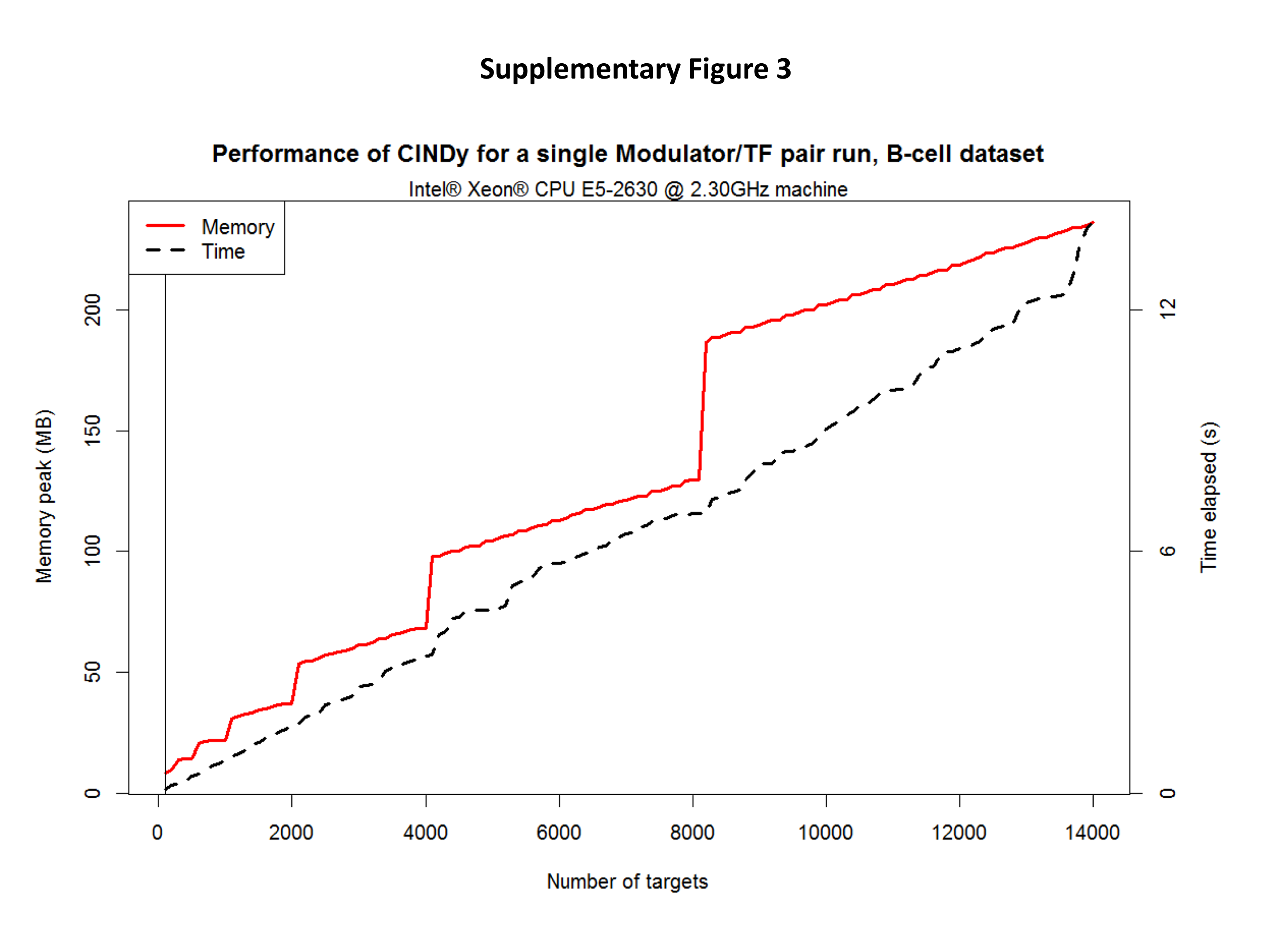

Supplement: Figure S3 — CINDy performance on a single TF-Modulator pair using increasing number of target genes. The vertical black line to the left indicates the average number of targets in the dataset (97.2). For this particular dataset, on average, MINDY using all genes is almost 130 times slower than using target genes, and requires almost 28 times more RAM. (TIF) [file pone.0109569.s003.tif]

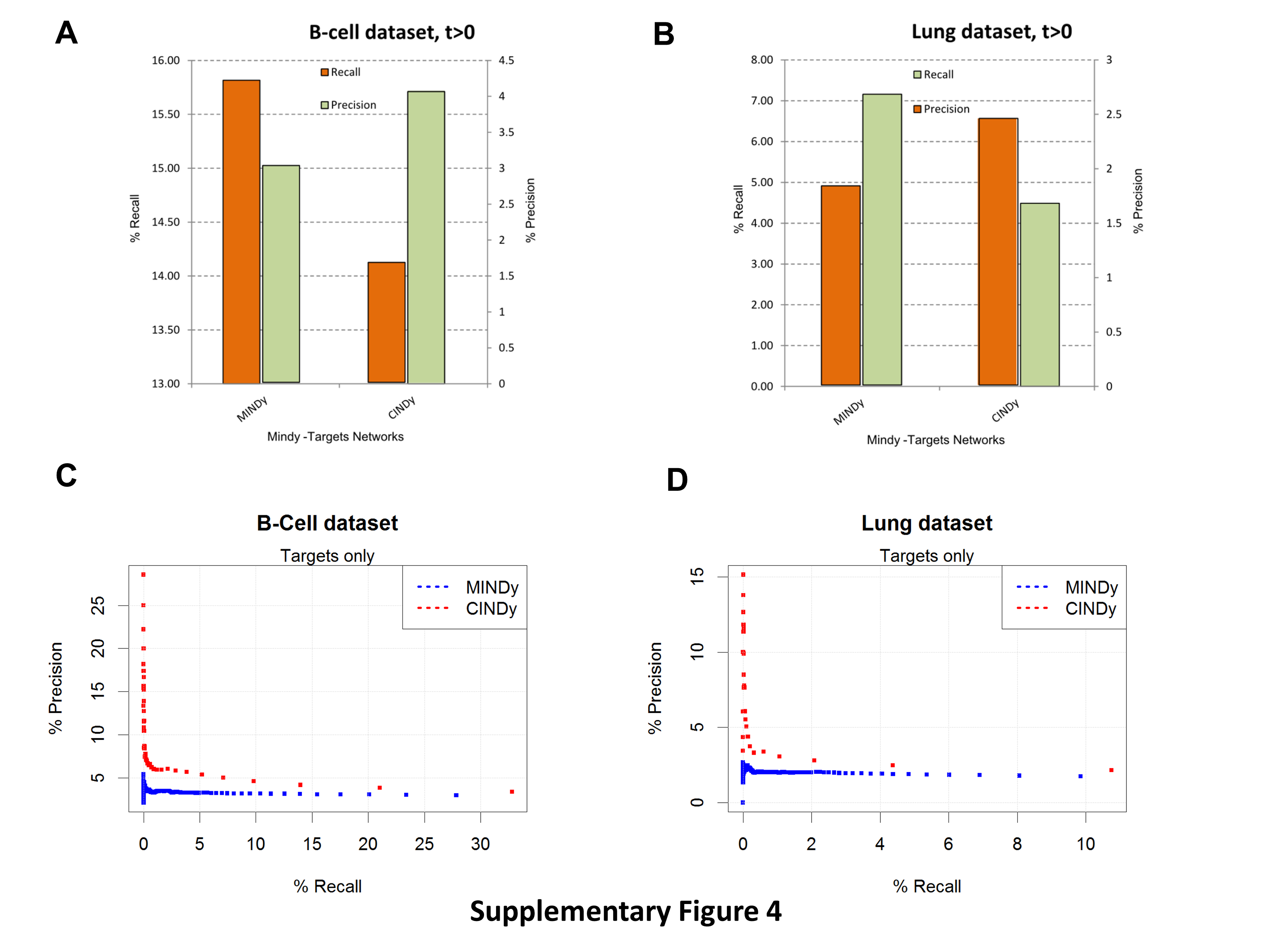

Supplement: Figure S4 — Benchmark of MINDy runs using a subset of target genes defined by ARACNe [27] (p-value 10e-8). A-B Precision and recall of MINDy, CINDy, intersection and union sets in the B-cell and Lung datasets, calculated over a golden set of four databases of experimentally validated PPIs between modulators and transcription factors. C-D Precision/Recall plots for MINDy (blue points) and CINDy (red points) at different robustness thresholds (see Materials and Methods ). (TIF) [file pone.0109569.s004.tif]

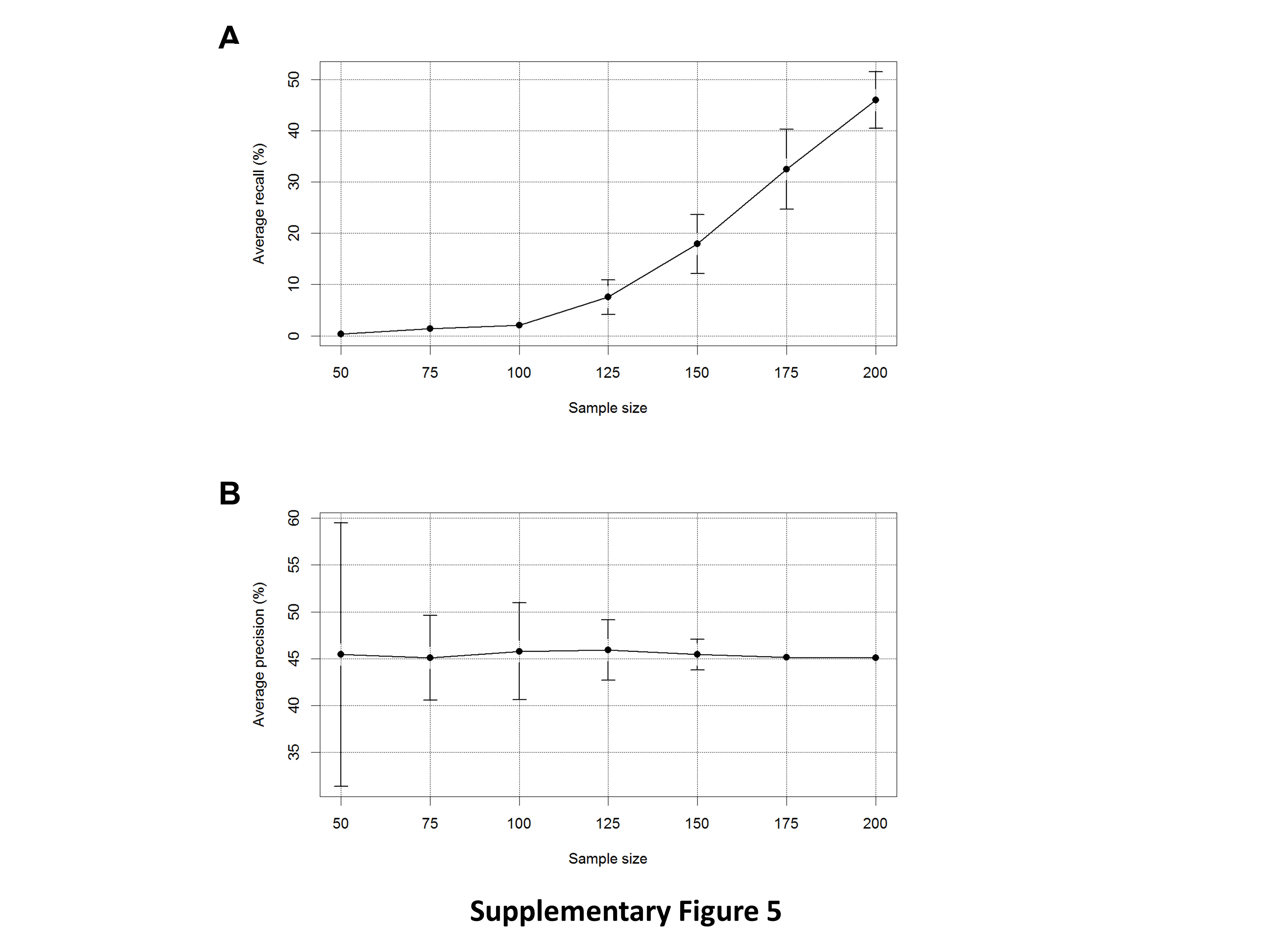

Supplement: Figure S5 — Effects of sample size on precision and recall in the B-cell dataset (226 samples). The precision/recall curves were calculated using the 100 TFs and modulators with most connections in the gold standard set (Figure S1). The error bars indicate the standard deviation in the estimation of precision and recall obtained by running CINDy over 100 datasets generated by subsampling. (TIF) [file pone.0109569.s005.tif]
